# A Novel Antihypertensive Pentapeptide Identified in Quinoa Bran Globulin Hydrolysates: Purification, In Silico Characterization, Molecular Docking with ACE and Stability against Different Food-Processing Conditions

**DOI:** 10.3390/nu14122420

**Published:** 2022-06-10

**Authors:** Yonghua Wei, Yongjuan Liu, Yan Li, Xian Wang, Yajun Zheng, Jianguo Xu, Shen Sang, Yuxi Liu

**Affiliations:** 1 College of Food Science, Shanxi Normal University, Taiyuan 030092, China; weiyonghua1983@126.com (Y.W.); liuyongjuan1984@126.com (Y.L.); liyan@sxnu.edu.cn (Y.L.); xssxsd2019@yeah.net (X.W.); lyx17535753828@163.com (Y.L.); 2 Yunnan Institute of Food Safety, Kunming University of Science and Technology, Kunming 650500, China; sangshen971104@163.com

**Keywords:** quinoa bran globulin hydrolysates, angiotensin-I-converting enzyme inhibitor, molecular docking, stability, security prediction in silico

## Abstract

The addition of food derived antihypertensive peptides to the diet is considered a reasonable way to prevent and lower blood pressure. However, data about stability of antihypertensive peptides against different food-processing conditions are limited. In this study, through Sephadex G-15 gel chromatography and RP-HPLC separation, UPLC-ESI-MS/MS analysis and in silico screening, a novel ACE-inhibitory pentapeptide Ser-Ala-Pro-Pro-Pro (IC_50_: 915.03 μmol/L) was identified in quinoa bran globulin hydrolysate. The inhibition patterns on angiotensin-I-converting enzyme and safety of SAPPP were studied using molecular docking and in silico predication, respectively. Results demonstrated that SAPPP could noncompetitively bind to active sites PRO519 and SER461 of ACE through short hydrogen bonds. SAPPP was resistant to different pH values (2.0–10.0), pasteurization conditions, addition of Na^+^, Mg^2+^, Fe^3+^ or K^+^, and the simulated gastrointestinal digestion. In contrast, SAPPP was unstable against heating at 100 °C for more than 50 min and the treatment of Zn^2+^ (5 mmol/L). These results indicated that peptides derived from quinoa globulin hydrolysates can be added into foods for antihypertension.

## 1. Introduction

Undesirable dietary habit is one of the main reasons for hypertension [1]. Improvement in diet habits including the addition of angiotensin-I-converting enzyme (ACE) inhibition peptides to the diet is considered a reasonable and practical way to prevent and lower blood pressure [2], since ACE is a key enzyme to elevate blood pressure in the body [3]. In the renin–angiotensin system, ACE can catalyze the conversion of angiotensin-I (an inactive decapeptide) into angiotensin-II of potential vasoconstriction effect, whereas ACE can inactivate the bradykinin that has vasodilatory activity in the kallikrein-kinin systems [4,5,6]. Compared with chemical synthesized ACE inhibitors, ACE-inhibitory peptides identified in food proteins have some advantages such as being economical, its abundant sources, its safety, and its easier acceptance by consumers [7]. Recently, technologies such as in silico screening, molecular docking and in silico simulated absorption and transportation have brought a more promising future for the utilization of ACE-inhibitory peptides in the food and other industries [8,9]. However, there are several challenges a new ACE-inhibitory peptide needs to face before it can be used in the food or pharmaceutical industry [10]. It has demonstrated that amino acid sequences, especially the *C*-terminal tripeptide, play a crucial role in physiological functions of ACE-inhibitory peptides [11]. However, the active sequence of ACE-inhibitory peptides can be degraded by proteases existing in the stomach and the intestine such as pepsin, trypsin and dipeptidase, thereby making the peptides inactive in vivo [12]. Moreover, the active sequence of ACE-inhibitory peptides can be changed under some processing conditions including acidic treatment, alkali treatment, heating, fermentation and interference of other food ingredients [13]. Moreover, safety is the first requirement for foods. Unsafe factors, especially potential toxicity and allergenicity, keep bioactive peptides out of recipes [10]. In addition, some physicochemical properties of peptides, such as isoelectric point, hydrophilicity and hydrophobicity, also can affect the utilization of peptides in specific food systems [14]. So, it is very necessary to investigate the security and physicochemical properties of ACE-inhibitory peptides, and stability against different food-processing conditions and gastrointestinal digestion.

Quinoa (*Chenopodium quinoa* Willd.) is an excellent plant protein resource for its high protein content (14–23 g/100 g) and high bioactivities such as anti-inflammatory, hypolipidemic and antioxidant activities [15,16,17,18]. Globulin accounts for 28.15 g/100 g quinoa protein [19]. The pre-experiment of this study demonstrated that the ACE-inhibitory activity of quinoa globulin hydrolysate was 29.41%, indicating that antihypertensive peptides can be isolated from it. Although several ACE-inhibitory peptides have been identified in quinoa albumin and quinoa yoghurt beverages [18,20], the security and stability against different processing conditions were not investigated. More importantly, to our best knowledge, few data about quinoa globulin ACE-inhibitory peptides are available. Quinoa bran is the major byproduct of quinoa powder milling and contains a high content of protein (around 24 g/100 g), but its utilization in the food industry is limited [19]. Therefore, the current study focuses on the identification, screening, characterization and security of ACE-inhibitory peptides from quinoa bran globulin hydrolysates using combined in silico and noncellular in vitro assays. Moreover, interactions between the peptides and ACE, stability profiles against different food-processing conditions and the gastrointestinal digestion are also investigated.

## 2. Materials and Methods

### 2.1. Materials

Quinoa bran was purchased from Wuzai New Country Mill, Xinzhou, China. Pepsin (from porcine stomach, 5 × 10^4^ U/g), Trypsin (from bovine pancreas, 5 × 10^4^ U/g), Papain (8 × 10^5^ U/g) and cellulase (from *Bacillus licheniformis*, 5 × 10^4^ U/g) were purchased from Tianjin Shengke Co., Ltd. (Tianjin, China). ACE and N-hippuryl-L-histidyl-L-leucine (HHL) were purchased from Sigma (St. Louis, MO, USA). Other chemicals were purchased from Yuhua Biochem. Co. (Taiyuan, China).

### 2.2. Preparation of Quinoa Bran Globulin Hydrolysates (QBGH)

As per the method of Zheng et al. [20] with slight modifications, quinoa bran was milled and passed through a sieve of 120 meshes. Quinoa bran powder was deoiled with *N*-hexane (1:25, *m*/*v*) in triplicate. Twenty grams of the defatted quinoa bran powder were dispersed in 400 mL of NaCl solution (0.25 mol/L), mixed thoroughly and stirred (180 r/min) in a stirring water bath at 40 °C for 80 min. The mixture was filtered with a filter paper and the filtrate was collected and centrifuged at 12,000× *g* for 12 min. The supernatant was dialyzed against deionized water (dH_2_O) with SP132590 dialysis membranes (3500 Da MWCO) at 4 °C for 24 h. Then, the dialysate solution was dried using FD1A50 freeze dryer (Kesheng Food Machinery Co., Zhucheng, China) to gain quinoa bran globulin (QBG).

Two grams of quinoa bran globulin powder was dissolved in 100 mL of phosphate buffer (0.1 mol/L, pH 7.4). Papain (100 U/g) was added and incubated in a stirring (180 r/min) water bath at 50 °C. The pH value of the reaction solution was maintained at pH 7.4 ± 0.1 by adjusting with 0.1 mol/L of NaOH at 30 min intervals. Two hours later, the reaction solution was heated at 100 °C for 8 min to inactivate the enzymes. After centrifugation at 13,500× *g* for 10 min, the supernatant was dried with the freeze drier to obtain quinoa bran globulin hydrolysates (QBGH). Moreover, the hydrolysis degree of QBGH was determined using trinitrobenzenesulfonic acid method [21].

### 2.3. ACE-Inhibitory Activity and Definition of IC_50_ Value

As per the same procedure described by Jimsheena and Gowda [22], inhibition activity on ACE was evaluated by comparing the quantity of produced hippuric acid before and after the addition of the peptides. IC_50_ value was calculated from the regression equation of ACE inhibition percentage versus different concentrations of the sample, and defined as the concentration of sample when ACE-inhibitory activity was 50%.

### 2.4. Purification and Identification of ACE-Inhibitory Peptides from QBGH

QBGH (1 mg/mL) was filtered utilizing an ultrafiltration membrane (0.45 μm) and the filtrate was purified on a Sephadex G-15 gel column (Φ1.6 × 100 cm) eluted by distilled water and monitored at 220 nm. The elution rate was 2.8 mL/min and the effluent fractions were collected 5 min intervals. The collected fractions were lyophilized and used for determination of ACE-inhibitory activity. The fraction of the highest inhibition capacity was further separated using reversed-phase high-performance liquid chromatography (RP-HPLC) with a NX-C_18_ column (Gemini, 110A, 4.6 × 250 mm, Phenomenex Co., Torrance, CA, USA). A linear gradient (2–80%, in 25 min) of acetonitrile containing 0.1% (*v*/*v*) of trifluoroacetate was used as mobile phase A, and deionized water containing 0.1% (*v*/*v*) of trifluoroacetate was used as mobile B. The flow rate was 1.0 mL/min and monitored at 220 nm. The subfractions were freeze-dried and used for ACE-inhibitory activity determination. Subfraction of the highest activity was used for peptide sequence identification.

The subfractions selected after the RP-HPLC was resolved in ultrapure water and then subjected to peptide sequence analysis. Analysis of peptide sequence was carried out using ultra-high-performance liquid chromatography (UPLC) coupled with electrospray ionization mass spectrometry (ESI-MS). The UPLC (U-3000 Series, Thermo Scientific, Waltham, USA) with an InfinityLab Poroshell 120 EC-C_18_ column (80 × 2.0 mm, 1.9 μm, Agilent Technologies, Santa Clara, CA, USA) was performed with gradient of acetonitrile (5–95%, 0–30 min; 95–5%, 30–35 min) as eluent A and ultrapure water (containing 0.1% formic acid) as eluent B was. The flow rate was 0.3 mL/min. The ESI-MS with a Q Exactive hybrid quadrupole-orbitrap mass spectrometer (Thermo Fisher, Bremen, Germany) was carried out with full MS 35000, ddMS^2^ 17500, AGC target value of 1 × 10^5^, and mass range of 120–1800 *m*/*z*. Moreover, the MS data were processed by De Novo™ software (Peak Studio 7.5, Bioinformatics Solutions, Inc., Waterloo, ON, Canada). The peptide sequences were matched to the published sequences of *Chenopodium quinoa* from the National Center for Biotechnology Information (NCBI, Bethesda, MD, USA) database.

### 2.5. Screening In Silico and Synthesis of the Selected Peptides

Peptide sequences identified in QBGH were analyzed utilizing the database BIOPEP (http://www.uwm.edu.pl/biochemia/index.php/en/biopep, accessed on 5 January 2021) and database AHTPDB (http://crdd.osdd.net/raghava/ahtpdb/, accessed on 7 January 2022) to find sequences of potential ACE inhibition activity and antihypertensive activity, respectively [23]. If the average local confidence (ALC) of a peptide sequence is greater than 85%, and its vector machine software scores (SVMS) is more than zero, it is acceptable as ACE-inhibitory peptide of potential antihypertension [7,24]. Some physicochemical properties of the selected peptides including isoelectric point, hydrophilicity, hydrophobicity and amphiphilicity were predicted using the database AHTPDB (http://crdd.osdd.net/raghava/ahtpdb/, accessed on 7 January 2022). Moreover, the chemical synthesis of selected sequence was conducted in Qiangyao Biotech Co. (Wuxi, China) with a standard solid-phase method.

### 2.6. Security Prediction In Silico

Potential toxicity of the peptides identified in QBGH was predicted using database ToxinPred (http://www.imtech.res.in/raghava/toxinpred/, accessed on 4 February 2022) [25]. Moreover, the potential allergenicity of the peptides was assessed using the database AlgPred (http://www.imtech.res.in/raghava/algpred/, accessed on 4 February 2022) with a threshold of −0.4 [26].

### 2.7. Molecular Docking

Molecular docking between ACE and the selected peptide sequences was carried out using the WYBYL-X2.11 software following the same procedure described by Ling et al. [27]. The three-dimensional (3D) crystal structure of ACE (PDB: 1O8A) was derived from the Protein Data Bank (http://www.rcsb.org/pdb/home/home.do, accessed on 19 January 2022). The obtained docking patterns between the peptide and ACE were selected mainly based on the T-scores (its least required thrust value is 6.0). Additionally, the C-scores, numbers and distance of hydrogen bonds formed between the active sites of ACE and the peptide sequence were also recorded.

### 2.8. Stability Profiles under Different Processing Conditions

#### 2.8.1. Thermal Stability Profiles

As per the method described by Zheng et al. [13], the synthesized peptide sequences identified in QBGH were dissolved in dH_2_O (100 μg/mL, pH 7.0) and then subjected to two thermal regimens. (1) The peptide solution was heated at different temperature to simulate pasteurization conditions: 63 °C, 30 min; 69 °C, 30 min; 72 °C, 15 s; 75 °C, 10 min; 80 °C, 25 s; and 100 °C, 12 min, respectively; and (2) the peptide solution was heated at 100 °C for 10, 20, 30, 40 and 50 min, respectively. After each treatment, peptide solutions were cooled to room temperature and then used for determination of ACE-inhibitory activity. Untreated peptides were used as control.

#### 2.8.2. pH-Stability Profiles

According to the method described by Chai et al. [28] with slight modifications, the synthesized peptide sequences identified in QBGH were dissolved in dH_2_O (100 μg/mL) and then separately adjusted to different pH values (pH 2–10). These solutions were incubated at 37 °C for 10 min and then all adjusted to pH 7.0. After that, these peptide solutions were used for determination of ACE-inhibitory activity with the untreated sample as comparison.

#### 2.8.3. Stability against Different Metal Ions

The stability against different metal ions was carried out as per the same procedure as described by Zheng et al. [13]. Briefly, peptide identified in QBGH was dissolved in dH_2_O (1 mg/mL). Aliquot of the peptide solution (100 μL) was subjected to the treatments of NaCl, KCl, MgSO_4_, ZnSO_4_ and FeCl_3_ (5 mmol/L, 100 μL), respectively. After incubation at 37 °C in a stirring bath (140 r/min) for 15 min, 50 μL of the reaction solution was taken and used for the determination of ACE-inhibitory activity. The remaining reaction solution was mixed with the same volume of ethanol (approximately 150 μL) and then centrifuged at 12,000× *g* for 10 min. The precipitate was collected, lyophilized and then mixed with dry KBr (1:50 m/m). The mix powder was ground, pelleted and analyzed using a 660-IR FTIR spectrometer (Varian, Palo Alto, CA, USA) with scanning range of 400 to 4000 cm^−1^ [13]. Peptides untreated were used as comparison.

### 2.9. Stability Profiles against Simulated Gastrointestinal Digestion

Following the method of Sun et al. [29], mixtures of trypsin (0.45 g), pig bile salt (3 g) and NaHCO_3_ (6.25 g) dissolved in 100 mL of dH_2_O (pH 6.8) were used to simulate intestinal fluid, whereas pepsin (40 mg) and NaCl (0.877 g) dissolved in 100 mL of dH_2_O (pH 2.0) were used as gastric fluid. Peptide solutions (dissolved in ultrapure water, 1 mg/mL) were incubated at 37 °C in a stirring bath (140 r/min) for 15 min; then, the simulated gastric fluid was added (gastric fluid: peptide solution = 1:10 *v*/*v*) and incubated at 37 °C (140 r/min) for 90 min. Then, the reaction solution was adjusted to pH 6.8 with Na_2_HPO_4_. The simulated intestinal fluid (the fluid: peptide solution = 1:10, *v*/*v*) was added and stirred (140 r/min) at 37 °C for 180 min. All the reaction solutions were heated at 100 °C for 8 min to inactivate the enzyme. Stability was calculated by comparing the ACE-inhibitory activities of the peptides before and after the digestion.

### 2.10. Data Analysis

Statistical analysis was performed using SPSS Version 16.0 software (Chicago, IL, USA). Data were expressed as mean ± standard errors (*n* ≥ 3), and one-way ANOVA was used to analyze variance.

## 3. Results and Discussion

### 3.1. Purification of ACE-Inhibitory Peptides from QBGH

**Figure 1:**
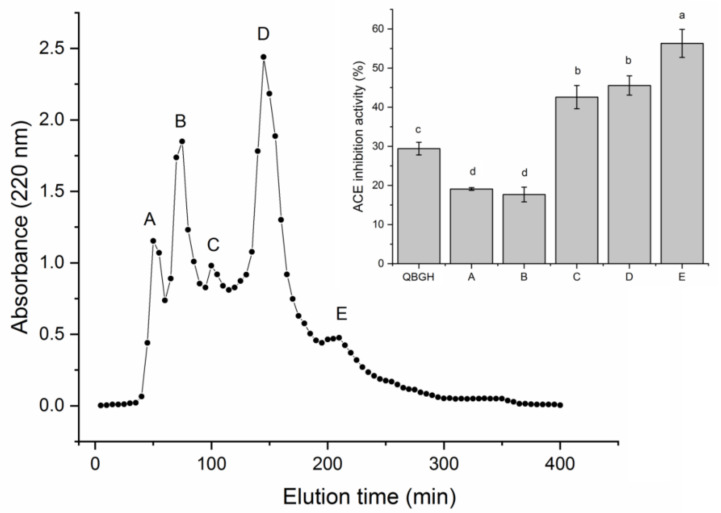
The separation profiles of quinoa bran globulin hydrolysate (QBGH) on Sephadex G-15 gel chromatography and angiotensin-I-converting enzyme (ACE) inhibitory activity of the subfractions. Uppercase letters (A–E) above the line and in the table represent the subfractions. Different lowercase letters (a–d) on the bars mean significant difference (*p* < 0.05).

**Figure 2:**
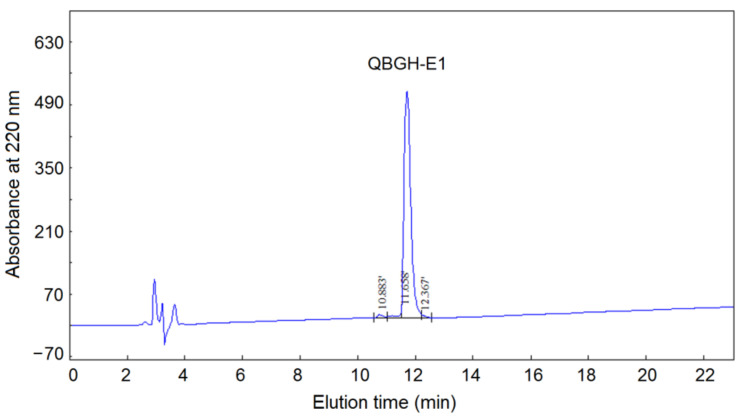
Reversed-phase high-performance liquid chromatography profile of subfraction QBGH-E

After the hydrolysis with Papain for 2 h, the ACE-inhibitory activity of quinoa bran globulin hydrolysate was 29.41% ± 1.62% (at 1 mg/mL), and the hydrolysis degree of quinoa bran globulin was 13.75% ± 2.25%. It has been demonstrated that Papain preferentially hydrolyzes peptide bonds linked with aromatic amino acid residues and Lys or Pro residues which can remarkably improve the ACE-inhibitory activity of peptides [5], so Papain was chose in the current study. As shown in Figure 1, through the Sephadex G-15 gel column chromatography, QBGH was separated into five major fractions (QBGH-A, QBGH-B, QBGH-C, QBGH-D and QBGH-E). Of these, QBGH-E showed the highest ACE-inhibitory activity (56.32% ± 3.58%, at 1 mg/mL), so it was further purified on reverse-phase high-performance liquid chromatography (RP-HPLC). As shown in Figure 2, a single main peak (QBGH-E1) with ACE-inhibitory activity of 58.88% ± 4.41% (at 1 mg/mL) appeared after the purification of QBGH-E on RP-HPLC. Thus, it was used for peptide sequence identification. In recent years, in silico screening of peptides using available databases makes identification and selection procedures of bioactive peptides faster, easier and more precise theoretically [8]. However, primary isolation and purification procedure is necessary to exclude most of the nontarget peptides and to improve the accuracy [30,31]. Therefore, a combined strategy including classic purification technologies (Sephadex gel and PR-HPLC chromatography) and in silico screening was utilized in the current study.

### 3.2. Peptide Identification from QBGH-E1 and In Silico Screening

**Figure 3:**
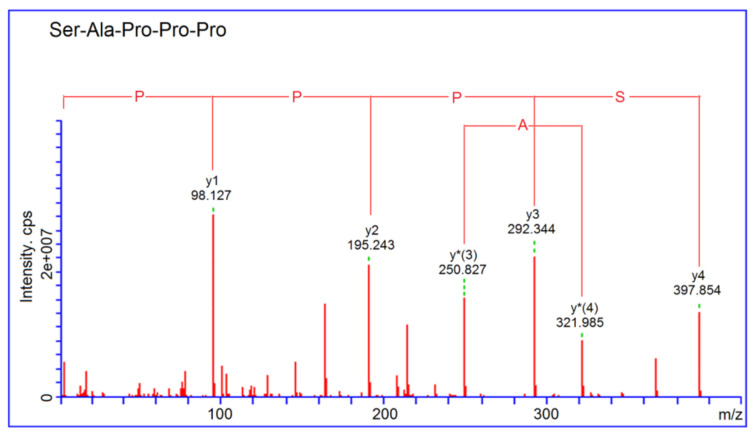
ESI-MS/MS spectra of SAPPP identified in quinoa globulin hydrolysates.

Based on the molecular mass and fragment information from the UPLC-ESI-MS/MS analysis, four oligopeptides of 4–9 amino acid residues were identified in QBGH-E1 (Table 1). The in silico screening result of these peptide sequences is shown in Table 1. Since the average local confidence (ALC) was greater than 85%, peptides Ser-Ala-Pro-Pro-Pro (467.57 Da) were demonstrated to have ACE-inhibitory activity [24]. Furthermore, SAPPP also had potential antihypertensive capacity since its vector machine software scores (SVMS) was more than 0.9 [7]. Therefore, SAPPP was chemically synthesized and the ESI-MS/MS spectra are shown in Figure 3.

**Figure 4:**
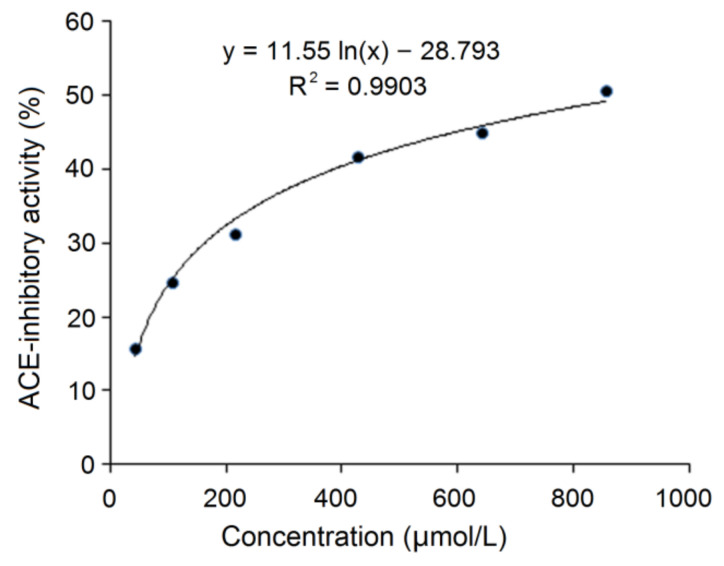
The regression analysis on ACE-inhibitory activity of SAPPP.

**Figure 5:**
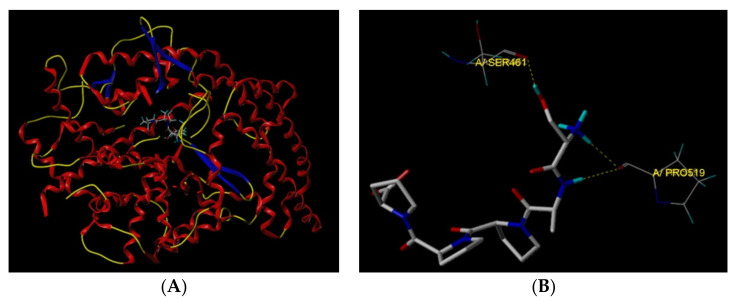
General overview (**A**) and local overview of the best-ranked docking pose (**B**) of SAPPP binding with ACE (PDB: 1O8A).

On the basis of the regression equation y = 11.55 ln(x) – 28.793 (R^2^ = 0.99) as shown in Figure 4, IC_50_ value of SAPPP was calculated to be 915.03 μmol/L (Table 1). Obviously, SAPPP was a pentapeptide of high content of Pro. Previous studies referring to the structure–activity relationship demonstrated that amino acid sequence, especially the *C*-terminal tripeptides, played a key role in ACE-inhibitory activity of peptides [12]. Some special acid residues including aromatic amino acids (Phe, Trp and Tyr) and Pro in the *C*-terminal tripeptides can tightly bind with active sites of ACE and show excellent ACE-inhibitory activity [7,32,33]. So, the Pro residue existing in *C*-terminal tripeptides of SAPPP was predominantly responsible for its high ACE-inhibitory activity. In addition, SAPPP (467.57 Da) showed less ACE-inhibitory activity than peptides GYGYNY (735.83 Da, IC_50_: 384 μmol/L) identified in camellia bran glutelin-2, SSYYPFK derived from oat naked globulin (890.4 Da, IC_50_: 91.82 μmol/L) and NMAINPSKENLCSTFCK identified in casein (IC_50_: 129.07 μmol/L) [13,20,34], which was inconsistent with the report that peptide mass is negatively related with the activity of ACE-inhibitory peptides [35]. The main reason was probably their different inhibition models on ACE as shown in Figure 5.

### 3.3. Prediction of Physicochemical Properties and Potential Security In Silico

Moreover, some physicochemical properties of SAPPP were predicted using the BIOPEP databases [23]. As shown in Table 1, the hydrophobicity of SAPPP was −0.04, consistent with the high content of hydrophobic amino acid residues (Ala and Pro). The amphiphilicity and hydrophilicity of SAPPP were 0.00 and −0.04, respectively, indicating that SAPPP can be used in both polar food systems and non-polar food systems [10]. Moreover, the isoelectric point of SAPPP was 5.88, suggesting that it should not be used in food systems of these pH values [13].

The result predicted by the database ToxinPred demonstrated that SAPPP has no potential toxicity (Table 1). However, the allergenicity of SAPPP was not predicted because only peptides of more than 12 amino acid residues can be analyzed in AlgPred (www.imtech.res.in/raghava/algpred/, accessed on 4 February 2022). A previous study reported that oligopeptides of smaller mass derived from foods were less allergenic in comparison with proteins of larger mass, because they usually do not contain the complete epitope [10]. However, a further study in vivo is still needed to clarify the security of SAPPP.

### 3.4. Molecular-Docking Analysis

Peptides can bind to the key binding pockets of ACE (S1, S_1_′, and S_2_′), and disturb the combination of ACE and the subunits (angiotensin-I or bradykinin), thereby showing a competitive inhibition pattern [32]. The result of molecular-docking analysis revealed that SAPPP can bind to two active sites of ACE (SER461 and PRO519) via three short hydrogen bonds (Figure 5B). Moreover, the docking T-Score of SAPPP (8.88) was higher than the required value (6.0). These results demonstrated that SAPPP had relatively strong binding power with ACE [27], which was also the main reason for the relatively high ACE-inhibitory activity of SAPPP (915.03 μmol/L). Moreover, both active sites SER461 and PRO519 do not belong to the key binding pockets of ACE (S1, S_1_′, and S_2_′), so SAPPP was a noncompetitive inhibitor of ACE. The noncompetitive inhibition pattern is the main reason why SAPPP showed lower ACE inhibition activity than competitive ACE-inhibitory peptides such as GYGYNY (IC_50_: 384 μmol/L) identified in camellia bran glutelin-2 and SSYYPFK derived from oat naked globulin (IC_50_: 91.82 μmol/L) [13,20].

### 3.5. Stability under Different Thermal Treatments

**Figure 6:**
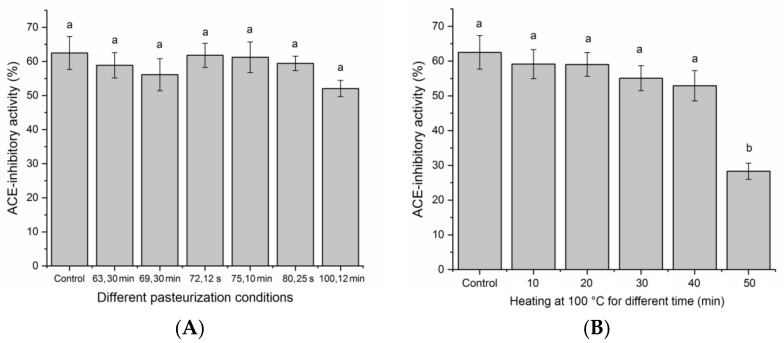
Stability of SAPPP (**A**) under different pasteurization conditions: heating at 63 °C, 30 min; 69 °C, 30 min; 72 °C, 15 s; 75 °C, 10 min; 80 °C, 25 s; and 100 °C, 12 min; (**B**) heating at 100 °C for 10–50 min. Control in each figure means untreated samples. Different lowercase letters (a–b) above the bars indicate significant differences (*p* < 0.05).

The stability of ACE-inhibitory peptides is one of the paramount influencing factors to determine the compatibility of peptides in different food systems, even bioavailability in vivo [28]. Heat treatment such as pasteurization and boiling are common processing techniques in the food industry. As shown in Figure 6A, the ACE-inhibitory activity of SAPPP was relatively stable against different pasteurization conditions including heating at 63 °C for 30 min, 69 °C for 30 min, 72 °C for 15 s, 75 °C for 10 min, 80 °C for 25 s and 100 °C for 12 min. Moreover, the result in Figure 6B demonstrated that SAPPP showed stable inhibition activity against heating at 100 °C for 40 min. When heating at 100 °C for 50 min, ACE-inhibitory activity of SAPPP was dramatically decreased (*p* < 0.05). These results suggested that SAPPP has excellent thermal stability. The main reason was the high content of Pro. Pro has been demonstrated to have high water-holding capacity and can confer plants excellent resistance to drought and heat [36]. 

### 3.6. Stability at Various pH Values

**Figure 7:**
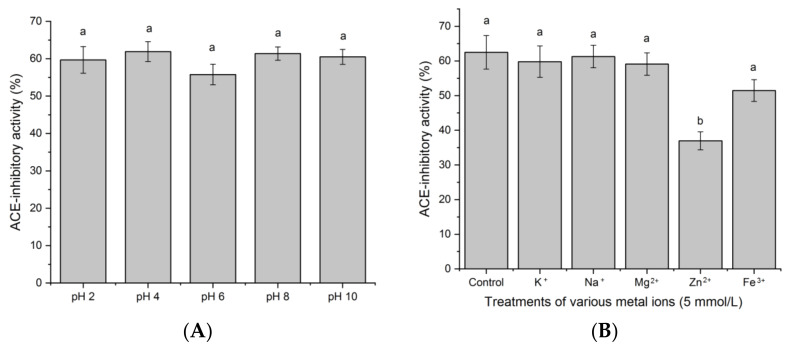
Stability of SAPPP (**A**) against different pH values; and (**B**) addition of different metal ions. Different lowercase letters (a–b) above the bars indicate significant differences (*p* < 0.05).

As shown in Figure 7A, there was no significant difference in ACE-inhibitory activity of SAPPP among the pH value range of 2.0–10.0. Although SAPPP showed a lower ACE-inhibitory activity at pH 6.0 (near its isoelectric point of pH 5.88, Table 1) than at other pH values, this difference was insignificant (*p* > 0.05). It is well recognized that the structure of a protein tends to shrink at isoelectric point, which may affect the interaction between ACE and peptides, resulting in a decrease in the ACE-inhibitory activity of peptides [14]. In general, ACE-inhibitory activity of SAPPP was resistant to various pH values.

### 3.7. Stability against Treatments of Different Metal Ions

As shown in Figure 7B, compared to the control (without metal ions), the ACE-inhibitory activity of SAPPP was not significantly different after the treatments of Na^+^, K^+^, Mg^2+^ and Fe^3+^ (*p* > 0.05), suggesting that SAPPP was resistant to the treatments of Na^2+^, K^+^, Mg^2+^ and Fe^3+^. In comparison, the ACE-inhibitory activity of SAPPP was remarkably decreased after the addition of Zn^2+^ (*p* < 0.05). The zinc ion is the key prosthetic group of ACE. So, addition of Zn^2+^ can change the polarity of microenvironment around the catalytic sites in the active center of ACE, thereby increasing the activity of ACE [7]. A similar trend was obtained by previous study [13]. 

### 3.8. Resistance to Gastrointestinal Digestion

**Figure 8:**
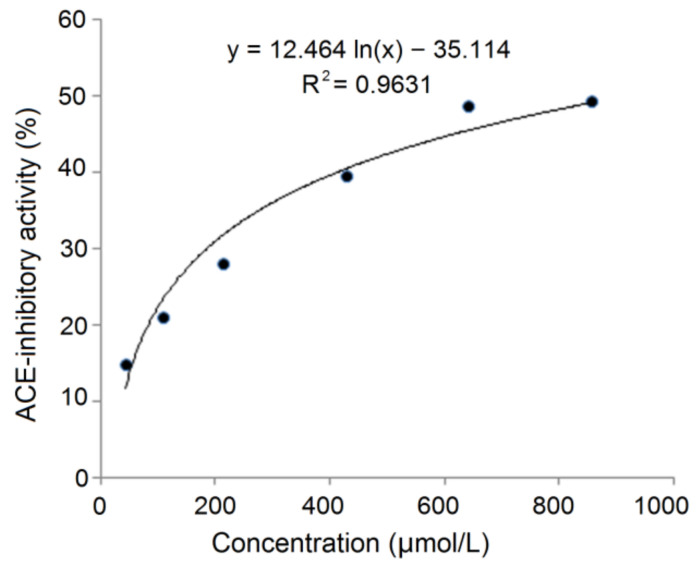
The regression analysis on ACE-inhibitory activity of SAPPP after the simulated gastrointestinal digestion.

The gastrointestinal digestion in vivo can cause degradation of peptide chains and make peptides inactive [37]. As shown in Figure 8, on the basis of the regression equation y = 12.464 ln(x) – 35.114 (R^2^ = 0.9603), the IC_50_ value of SAPPP after the digestion was calculated to be 926.00 μmol/L (Table 1). This value is higher but not significantly than that of untreated SAPPP (915.03 μmol/L, Figure 4) (*p* > 0.05). These results indicated that the ACE-inhibitory activity of SAPPP was relatively stable during the simulated gastrointestinal digestion. The main reason was the structural characteristics of SAPPP. It is well recognized that pepsin and trypsin in the gastric and the intestinal region prefer to hydrolyze amide bonds binding with aromatic amino acid residues (Tyr, Phe and Trp), Arg and Lys residues [12,35]. Thus, peptides rich in aromatic amino acids, Arg and Lys residues are susceptible to gastrointestinal digestion [29]. However, SAPPP does not contain these amino acid residues. More importantly, the Pro residues existing in SAPPP were the main reason for its resistance to gastrointestinal digestion. An increasing number of studies have demonstrated that Pro residue can effectively improve the resistance of peptides to digestion by pepsin and trypsin [32]. Pro is recognized as a ‘rigid amino acid’ and can confer plants excellent resistance to drought and heat [36]. Therefore, the accumulation of Proline has been widely found in drought-tolerant and cold-tolerant plants such as quinoa, naked oat and millet [13,20,38]. Proline-rich peptides including ELHPQ, LHPQ and KPVPR identified in canary seed and SSYYPFK identified in naked oat also exhibited excellent stability against gastrointestinal digestion [7,20]. The influence of the simulated gastrointestinal digestion on peptide sequences of SAPPP needs to be further investigated.

## 4. Conclusions

A novel ACE-inhibitory peptide SAPPP (IC_50_: 915.03 μmol/L) without potential toxicity was identified in quinoa bran globulin hydrolysates. SAPPP could noncompetitively bind to active sites PRO519 and SER461 of ACE through short hydrogen bonds. SAPPP was resistant to different pH values (2.0–10.0); pasteurization conditions; treatments of Na^+^, Mg^2+^, Fe^3+^ or K^+^; and the simulated gastrointestinal digestion. In contrast, SAPPP was unstable against heating at 100 °C for more than 50 min, and the treatment of Zn^2+^ (5 mmol/L). These results indicated that SAPPP derived from quinoa bran could be used as an ingredient of antihypertensive foods. In order to validate the results presented in this paper, more non-celular and particularly celular assays, should be performed.

## Figures and Tables

**Table 1:** Amino acid sequence, in silico screening of ACE-inhibitory and antihypertensive activity, in silico prediction of physicochemical properties, toxicity and allergenicity of peptides identified in quinoa bran globulin hydrolysates by UPLC-ESI-MS/MS.

Peptide Sequence	ICCPIIAKMY	MGAAAGM	SAPPP
Mass (Da)	469.66	933.19	467.57
Matched sequence in *Chenopodium quinoa* ^a^	G.ICCPIIAKMY.P	A.GMGAAAGM.D	V.SAPPP.L
ALC (%)	72	76	91
SVMS	−0.02	−0.95	1.16
Prediction	Non-AHT	Non-AHT	AHT
Hydrophilicity ^b^	−0.85	−0.59	−0.04
Amphiphilicity	0.87	0.00	0.00
Hydrophobicity	0.16	0.23	−0.04
Isoelectric point	8.36	5.88	5.88
IC_50_ of ACE-inhibitory activity (μmol/L)	ND	ND	915.03
IC_50_ of ACE-inhibitory activity after gastrointestinal digestion (μmol/L)	ND	ND	926.00
Toxicity ^c^	Non-Toxin	Non-Toxin	Non-Toxin
Allergenicity	ND	ND	ND

^a^ From National Center for Biotechnology Information (NCBI). ALC (average local confidence) was calculated using published data in BIOPEP database; SVMS: vector machine software score; and AHT: antihypertension. ^b^ Physicochemical properties were predicted using the AHTPDB database. ^c^ The potential toxicity and allergenicity were predicted using the database ToxinPred (www.imtech.res.in/raghava/toxinpred/, accessed on 4 February 2022) and AlgPred (www.imtech.res.in/raghava/algpred/, accessed on 4 February 2022), respectively. ND: not measured.

## Data Availability

Not applicable.
